# BIODEMOGRAPHY OF AGING, CELEBRATING JAMES W. VAUPEL, PHD

**DOI:** 10.1093/geroni/igad104.0195

**Published:** 2023-12-21

**Authors:** Kaare Christensen

## Abstract

James W. Vaupel, PhD (1945 – 2022), was an international leader in demography and aging research and a pioneer in the field of biodemography. He was a highly creative researcher and very entrepreneurial in the development of new interdisciplinary research environments. This symposium highlights a few of the biodemographic research areas that Dr. Vaupel has been a catalyst for. Denmark was Dr. Vaupel’s home country during the last third of his life, and Dr. Christensen reports on findings from Danish nationwide genetic-epidemiological studies on twins and the oldest old: familial influence on aging phenotypes, cohort differences in health among the oldest old, associations of early life events with late life health, as well as tongue-in-cheek research on perceived age and teeth. Dr. Alberts discusses the ‘invariant rate of aging’ hypothesis that was developed by Vaupel and others. She presents comparative analyses from multiple nonhuman primate populations showing that, while life expectancy can continue to improve, we probably can’t slow the demographic rate of aging. Dr. Campos presents evidence from a wild baboon population in Kenya that glucocorticoid levels—biological markers of stress responses—are strong prognostic indicators of survival and may be key explanations of life span disparities. Dr. Carey presents key discoveries from large-scale studies involving the Mediterranean fruit fly. These include the slowing of mortality at older ages, context-specific sex mortality differentials, a behavioral biomarker for morbidity, dietary conditions for maximizing lifespan versus reproduction and a stationary population identity whereby life lived equals life left.

